# Adhesive Gold Foil

**Published:** 1858-07

**Authors:** 


*Adhesive Gold Foil.*—Dr. A. M. Leslie, formerly a practitioner of Dentistry of Cincinnati, Ohio, but now the proprietor of a Dental Depot in St. Louis, Mo., recently sent to the senior Editor a few leaves of adhesive gold foil, manufactured in his establishment from the scraps of leaf gold without remelting. It is a beautiful article, and when annealed may be welded one piece to another with the greatest facility with filling instruments having integrating points. The crown or a part of a crown of a tooth may be as readily built up with it as with crystal or sponge gold, by a dexterous operator. We take pleasure, therefore, in calling the attention of the profession to it.

